# Combination Therapy Potential of HTD1801 (Berberine Ursodeoxycholate) in MASLD/MASH and Cardiovascular–Kidney–Metabolic Disease: A Narrative Review

**DOI:** 10.3390/ph19101572

**Published:** 2026-10-03

**Authors:** Boknyun Kim, Joseph Kim, Grace Kim, Kitai Kim

**Affiliations:** 1 Virginia Headquarters Campus, Virginia University of Integrative Medicine, 1980 Gallows Road, Vienna, VA 22182, USA; bkim251@vuim.edu (B.K.); kkim20@vuim.edu (K.K.); 2 Greater Los Angeles Science Foundation, Los Angeles, CA 90405, USA; boknyun-kim@losangelesscience.org (B.K.); joseph-kim@losangelesscience.org (J.K.); grace-kim@losangelesscience.org (G.K.); kitai-kim@losangelesscience.org (K.K.); 3 Human Stem Cell and Genome Engineering Center, University of California, Los Angeles, CHS 36-125/133/143 CHS38-133, 650 Charles E Young Dr S, Los Angeles, CA 90095, USA; 4 Thomas C. Jenkins Department of Biophysics, Johns Hopkins University, 110 Jenkins Hall, 3400 N. Charles Street, Baltimore, MD 21218, USA; jkim671@jhu.edu; 5 Department of Computer Science, Johns Hopkins University, 160 Malone Hall, 3400 N. Charles Street, Baltimore, MD 21218, USA; jkim671@cs.jhu.edu; 6 Life Sciences Department, Santa Monica College, 1900 Pico Boulevard, Santa Monica, CA 90405, USA; kim_grace_hyelim01@student.smc.edu

**Keywords:** HTD1801, berberine ursodeoxycholate, combination therapy, metabolic dysfunction-associated steatotic liver disease, metabolic dysfunction-associated steatohepatitis, type 2 diabetes mellitus, cardiovascular–kidney–metabolic syndrome

## Abstract

**Background/Objectives:** The cardiovascular–kidney–metabolic (CKM) continuum is characterized by the close interrelatedness of metabolic, hepatic, cardiovascular, and renal disease, making multidomain therapies particularly relevant to MASLD/MASH. HTD1801, an investigational ionic therapeutic, combines berberine’s metabolic effects with the hepatoprotective effects of ursodeoxycholic acid (UDCA), making it a potential multidomain therapy within this setting. This review had two related goals: first, to synthesize the mechanistic and clinical evidence for HTD1801 itself; and second, to evaluate the rationale and available evidence for extending this complementary-action approach through combination with other therapies relevant to MASLD/MASH and CKM disease. **Methods:** PubMed/MEDLINE, ClinicalTrials.gov, conference abstracts, and selected regulatory or sponsor sources were reviewed through 9 August 2026. Six CKM-relevant therapeutic classes were selected for their human evidence of multidomain benefit: metformin, GLP-1 receptor agonists, statins, SGLT2 inhibitors, PPAR agonists, and resmetirom. Studies of both HTD1801 and its individual components, berberine and UDCA, were included. A systematic review or meta-analysis was not feasible due to the limited and heterogeneous evidence base, instead necessitatinganarrative review. **Results:** Across seven completed HTD1801 clinical studies with publicly reported results, treatment showed multidomain metabolic and hepatic benefits across the CKM spectrum, including improvements in HbA1c, LDL-C, MRI-PDFF hepatic fat, ALT, AST, and GGT. Direct human evidence: HTD1801 + metformin, supported by a Phase 3 randomized add-on trial. Indirect human evidence: HTD1801 + SGLT2 inhibitors. Preclinical evidence: HTD1801 + GLP-1 receptor agonists; berberine + statins. Theoretical mechanistic rationale only: HTD1801 + PPAR agonists; HTD1801 + resmetirom. Overall, mechanistic and preclinical rationale has advanced further than direct human validation, and evidence remains centered largely on surrogate rather than definitive clinical outcomes. **Conclusions:** HTD1801 has demonstrated multidomain metabolic and hepatic activity, but direct human evidence supporting its use with other CKM therapies remains limited and is currently established only for metformin. The principal next step is to test HTD1801 in dedicated combination RCTs for clinically meaningful benefit. Further studies should characterize combination-specific safety and tolerability and compare HTD1801 with separate berberine and UDCA administration.

## 1. Background

Metabolic dysfunction-associated steatotic liver disease (MASLD) and its inflammatory subtype, metabolic dysfunction-associated steatohepatitis (MASH), are closely associated with obesity and type 2 diabetes mellitus (T2DM) [1,2], and their global burden has increased substantially in recent years [3,4]. Rather than representing isolated liver disorders, MASLD and MASH should be viewed within the broader cardiovascular–kidney–metabolic (CKM) syndrome framework, in which metabolic dysfunction, chronic kidney disease, cardiovascular disease, and hepatic steatosis are interrelated and mutually reinforcing [5]. Accordingly, management of MASH in individuals with T2DM should extend beyond liver-directed care alone and address the broader metabolic and cardiorenal context [6]. Treating MASLD or MASH as purely hepatic disorders overlooks the close interactions among metabolic, cardiovascular, renal, and hepatic pathways.

Although current guidelines recommend effective therapies for individual components of CKM syndrome, no single agent comprehensively addresses metabolic, hepatic, and cardiorenal risk simultaneously. This review therefore focuses on six therapeutic classes—metformin, glucagon-like peptide-1 (GLP-1) receptor agonists, statins, sodium-glucose cotransporter-2 (SGLT2) inhibitors, peroxisome proliferator-activated receptor (PPAR) agonists, and resmetirom. These classes were selected because each has published human clinical evidence relevant to at least one metabolic or cardiorenal outcome and one hepatic outcome, allowing comparison of their respective strengths and limitations across the CKM spectrum.

Independently of HTD1801, GLP-1 receptor agonists and SGLT2 inhibitors provide important cardiovascular and renal benefits, including reductions in major adverse cardiovascular events, cardiovascular death, and progression of chronic kidney disease [7,8]. For example, in EMPA-REG OUTCOME, empagliflozin reduced three-point major adverse cardiovascular events by 14% and cardiovascular death by 38% in patients with T2DM and established atherosclerotic cardiovascular disease [8,9]. Semaglutidehas also demonstrated histologic benefit in MASH [10]. Nevertheless, the extent to which incretin-based therapies provide durable fibrosis regression and comprehensive hepatic benefit across the broader CKM syndrome continuum remains under investigation [7]. Conversely, liver-directed therapies such as PPAR agonists and resmetirom can improve histologic endpoints [11,12], but definitive evidence of major cardiovascular or kidney outcome benefits remains limited. Foundational therapies, including metformin and statins, remain essential for glycemic and cardiovascular risk management but rarely reverse established liver injury.

HTD1801 was developed as an ionic salt of berberine and ursodeoxycholic acid (UDCA), intended to integrate their potentially complementary pharmacologic actions within a single therapeutic entity rather than as a simple physical mixture. Ionic complexation has also been proposed as a formulation strategy to improve the solubility and oral bioavailability of berberine, whose clinical utility is limited by poor intestinal absorption and extensive first-pass elimination [13]. This review examines the mechanistic profile of HTD1801 and its potential intersections with the six therapeutic classes discussed above.

## 2. Methods

For this narrative review, we examined literature indexed in PubMed/MEDLINE through 9 August 2026, focusing on studies that directly evaluated HTD1801 (HighTide Therapeutics, Shenzhen, China) or its individual components, berberine and ursodeoxycholic acid (UDCA). Because HTD1801 has a relatively limited evidence base, the search strategy was intentionally broad. Keywords included “HTD1801,” “berberine ursodeoxycholate,” “BUDCA,” “berberine,” “ursodeoxycholic acid,” “MASLD,” “MASH,” and “type 2 diabetes,” along with names of therapeutic classes commonly used in MASLD/MASH and CKM disease: metformin, GLP-1 receptor agonists, statins, SGLT2 inhibitors, PPAR agonists, and resmetirom. Studies were included if they evaluated HTD1801 directly or evaluated its individual components (berberine or UDCA) in a manner relevant to the mechanistic or clinical questions addressed here; studies of unrelated compounds, or of berberine/UDCA in contexts with no plausible relevance to HTD1801, were excluded.

Eligibility criteria were specified before screening and applied in two stages: title and abstract screening, followed by full-text assessment. Sources were included if they (i) evaluated HTD1801 (berberine ursodeoxycholate) directly, or evaluated berberine or UDCA individually in a manner relevant to the mechanistic or clinical questions addressed here; (ii) reported at least one metabolic, hepatic, cardiovascular, or renal outcome, or a mechanistic finding relevant to these domains; (iii) were human clinical studies (randomized or non-randomized), systematic reviews or meta-analyses, or preclinical studies providing mechanistic support for observations in humans; (iv) were written in English; and (v) were available as peer-reviewed publications, registered trial records, regulatory or sponsor communications, or abstracts presented at ADA, EASD, EASL, or ERA meetings. Clinical practice guidelines were additionally included where needed to frame current standards of care.

**Figure 1:**
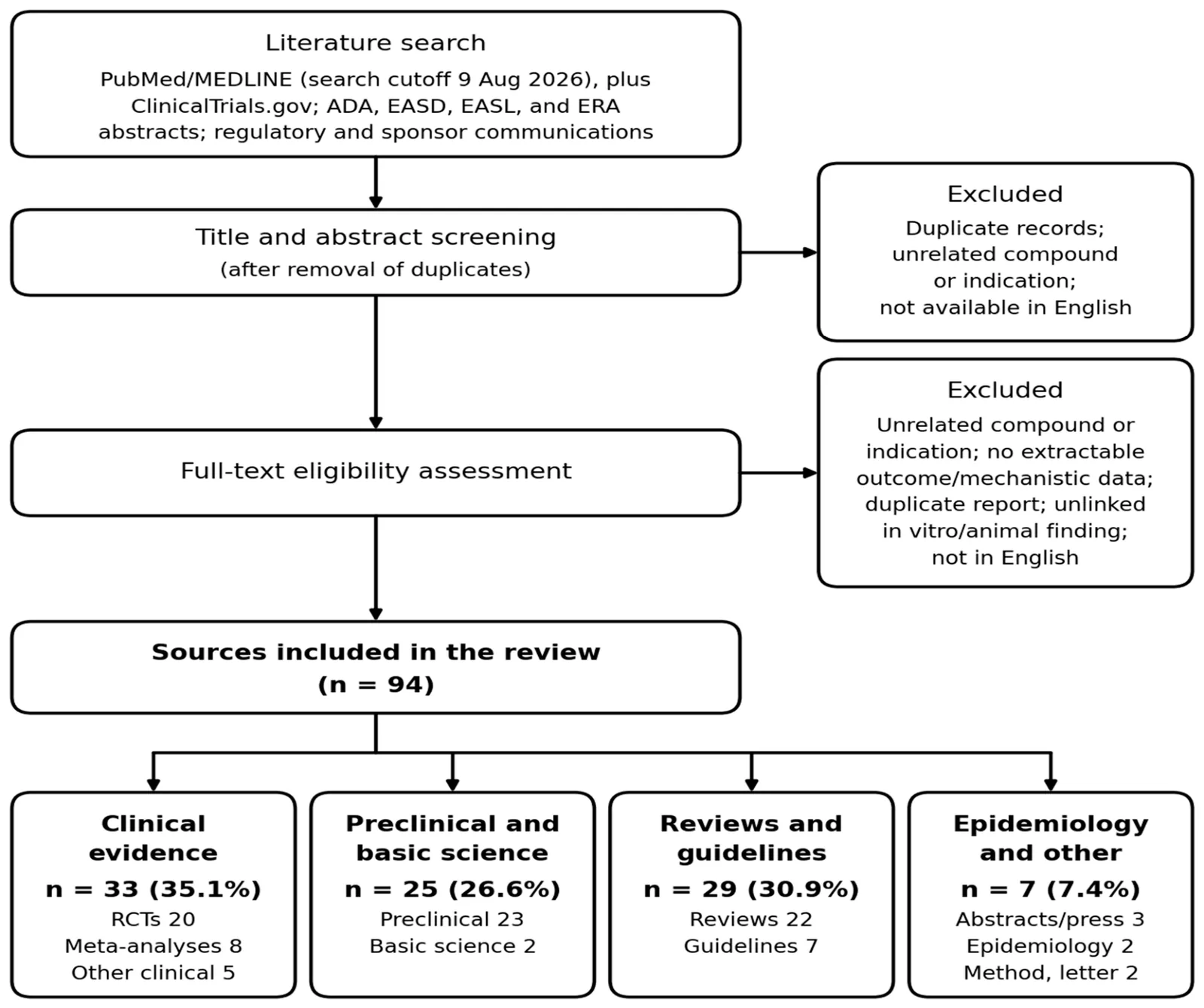
Selectionprocess for this narrative review. Because this is a narrative rather than a systematic review, the diagram summarizes the sequence of identification, screening, and eligibility assessment and the reasons sources were excluded at each stage, rather than reporting a PRISMA-style numeric flow; only the final number of included sources (*n* = 94) is reported. ADA, American Diabetes Association; EASD, European Association for the Study of Diabetes; EASL, European Association for the Study of the Liver; ERA, European Renal Association; UDCA, ursodeoxycholic acid.

Sources were excluded if they (i) evaluated unrelated compounds, or berberine or UDCA in contexts with no plausible relevance to HTD1801, such as oncologic, antimicrobial, or dermatologic applications; (ii) provided no extractable outcome or mechanistic data, including editorials, commentaries, and narrative overviews without primary data; (iii) were not available in English; (iv) were duplicate reports of a trial already represented by a more complete source, in which case the most recent and most complete peer-reviewed version was retained; or (v) reported in vitro or animal findings that could not be linked to a pathway discussed in this review. No restriction on publication year was applied. Screening and eligibility assessment were carried out by one author and verified by a second, with disagreements resolved by discussion. The selection process is summarized in Figure 1; because stage-by-stage counts were not retained, only the final number of included sources (*n* = 94) is reported.

We also reviewed ClinicalTrials.gov (https://clinicaltrials.gov, accessed on 9 August 2026), last checked on 9 August 2026, to identify ongoing, completed, or unpublished studies. Abstracts from recent ADA, EASL, and ERA meetings were incorporated when they provided mechanistic or early clinical insights not yet available in peer-reviewed publications. Sponsor-released materials were used sparingly and only when they clarified trial design, regulatory status, or endpoints; such evidence is identified as preliminary or non-peer-reviewed. When the same trial was reported in more than one source (for example, a conference abstract, a sponsor press release, and a subsequent peer-reviewed article), the most recent and most complete peer-reviewed version was used as the primary reference for that trial throughout the review, and earlier reports were cited only where needed to document the preliminary status of the data at an earlier time point.

Because the available studies differ substantially in design, duration, and patient characteristics, and because published human biopsy-based efficacy results for HTD1801 were unavailable at the search cutoff, a formal meta-analysis or structured risk-of-bias assessment was not appropriate. Instead, we adopted a narrative approach, interpreting each study within its own context rather than forcing heterogeneous data into a single analytical framework. Findings from berberine or UDCA monotherapy were included only when they helped explain mechanistic pathways relevant to HTD1801; these component-level insights are distinguished from HTD1801-specific evidence throughout the review.

Several methodological limitations affect interpretation of the key trials summarized in Table 1, independent of their heterogeneity. Most completed studies were short in duration (12–24 weeks, with longer-term data available only from open-label extensions), relied on surrogate rather than histologic endpoints (HbA1c, MRI-PDFF, LDL-C), and, for the mechanistic and safety-focused Phase 1b/2a studies, enrolled small samples (*n*= 48–113). At the time of this review, the HARMONY and SYMPHONY-1 trials were available only as conference presentations or sponsor press releases, and the SYMPHONY-1 primary results have since been published in a peer-reviewed journal (Diabetes Care), while HARMONY remains conference/press-release only. Finally, all completed trials to date have been sponsor-funded, which may influence outcome selection and reporting and underscores the need for independent replication.

Conference abstracts and other non-peer-reviewed sources were treated as preliminary signals. They can provide useful early information, but they require cautious interpretation until full peer-reviewed data become available. This approach allowed the review to remain current without overstating the certainty of early findings.

## 3. Mechanisms of Action

HTD1801 dissociates in the gastrointestinal tract into berberine and ursodeoxycholic acid (UDCA), and much of what is currently known about its biology comes from studies of these individual components rather than the intact ionic complex.

**Figure 2:**
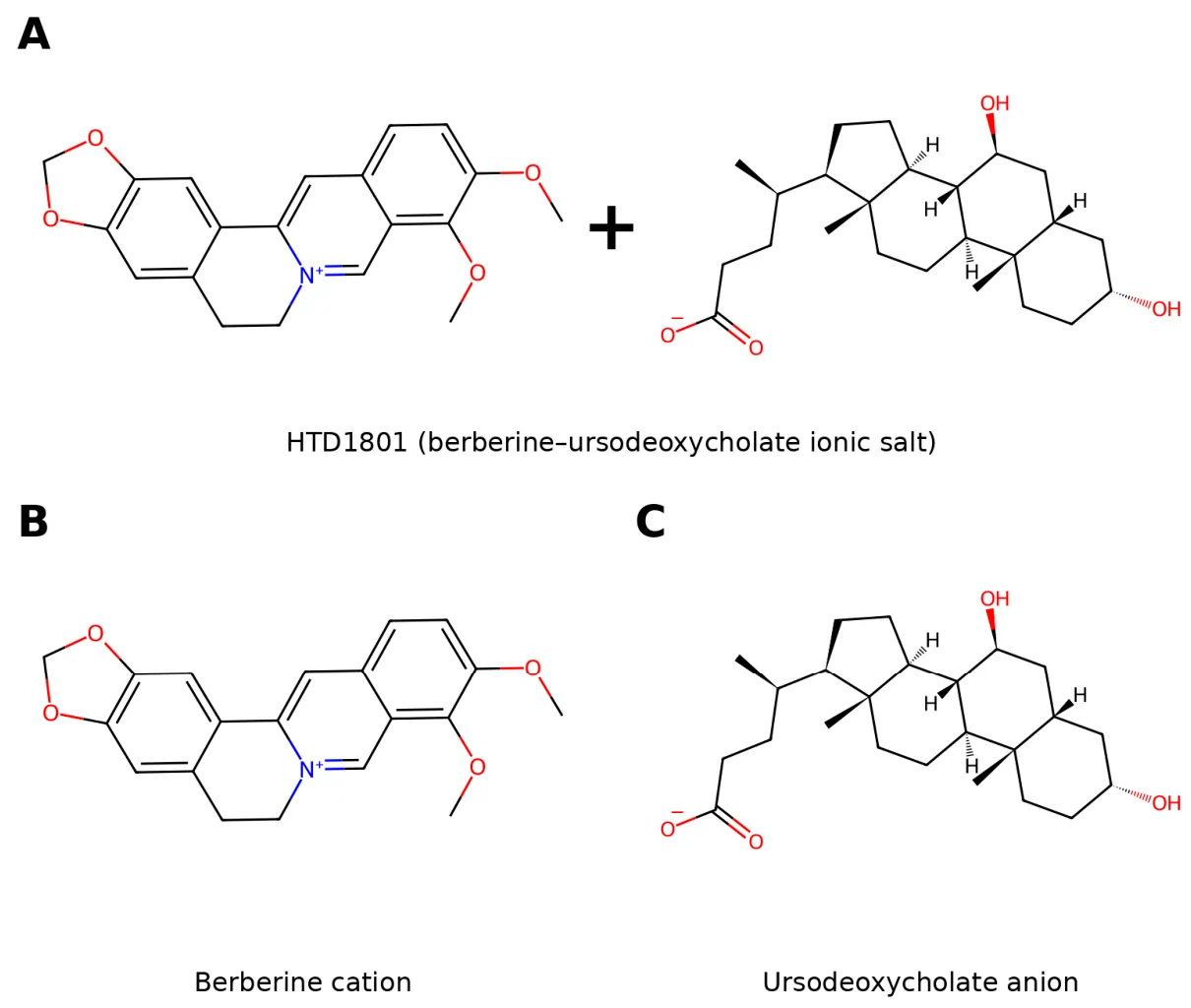
Chemical structures of HTD1801 and its constituent ions. (**A**) HTD1801, the ionic salt formed between the berberine cation and the ursodeoxycholate anion. (**B**) Berberine cation, a permanently charged quaternary-ammonium alkaloid with no neutral free-base form. (**C**) Ursodeoxycholate anion, the ionized form of ursodeoxycholic acid (UDCA) predominating at intestinal pH. Structures were rendered from PubChem reference SMILES (RDKit) using default coloring conventions (red: oxygen-containing functional groups; blue: nitrogen-containing functional groups).

Berberine is a permanently charged quaternary-ammonium alkaloid with no neutral free base form (Figure 2); it is pharmaceutically supplied only as salts (chloride, sulfate) or lipid complexes, and its low, saturable intestinal absorption and extensive CYP-mediated hepatic metabolism are believed to be broadly similar regardless of the specific salt used, although direct head-to-head comparative data across berberine salts remain limited [21,22]. UDCA, by contrast, is a weakly acidic bile acid whose ionization state—and therefore solubility—shifts with intestinal pH, and it undergoes standard hepatic conjugation and enterohepatic recirculation [23]. These differing absorption and clearance pathways are relevant to combination potential because they determine which co-administered drug classes are most likely to compete for shared transporters or metabolic enzymes, as discussed in later sections. No independent physicochemical data for the HTD1801 ionic complex itself (versus its separate components) are currently available in the literature. In the acidic gastric environment, the berberine–UDCA ionic bond is expected to dissociate rapidly, effectively generating berberine hydrochloride in situ; the resulting exposure is therefore expected to approximate that of orally administered berberine hydrochloride, whose plasma pharmacokinetics have been characterized in rats [24]. Potential CYP-mediated interactions with several combination partners are addressed in later sections. Whether meaningful pre-gastric (oral-mucosal) absorption of berberine occurs has not been studied and is noted here only as an unresolved gap, not a mechanism expected to materially affect combination outcomes. Whether the two components mutually influence each other’s hepatic enzyme-mediated biotransformation has likewise not been directly examined and remains an open question for HTD1801.

Berberine’s metabolic actions have been characterized for decades. By inhibiting mitochondrial complex I, berberine increases the intracellular AMP:ATP ratio and activates AMP-activated protein kinase (AMPK) [25]. Activation of AMPK suppresses hepatic gluconeogenesis, slows de novo lipogenesis [26,27], and promotes autophagy [28]. These pathways help explain why berberine consistently reduces hepatic fat and improves systemic metabolic balance. Although these mechanisms are well documented for berberine monotherapy, they have not yet been directly confirmed for HTD1801 itself.

UDCA acts primarily on the hepatocellular stress axis. It reduces endoplasmic reticulum stress, prevents mitochondrial permeability transition pore opening, and interferes with Bax-mediated apoptosis [29]. In broad terms, berberine tends to correct metabolic dysfunction, whereas UDCA protects hepatocytes under cellular stress. In a phase 2 clinical trial of HTD1801, the clearest evidence of improved insulin homeostasis was observed for the homeostatic model assessment of insulin resistance (HOMA-IR), with a numerically greater reduction at the higher dose: least-squares mean changes from baseline were −0.3 (SE, 0.3) with 500 mg twice daily and −0.9 (SE, 0.3) with 1000 mg twice daily, compared with +0.5 (SE, 0.3) with placebo (*p* = 0.13 and *p* = 0.006, respectively; *n* = 33–34 per group) [14]. While encouraging, HOMA-IR reflects systemic insulin sensitivity and cannot be taken as direct evidence of hepatic AMPK activation.

Berberine has also been associated with anti-inflammatory effects in preclinical models, particularly through suppression of NF-κB and NLRP3 inflammasome signaling, resulting in reductions in proinflammatory cytokines such as tumor necrosis factor-α (TNF-α), and interleukin-1β (IL-1β) [30,31]. Whether HTD1801 reproduces these anti-inflammatory effects in humans remains uncertain. UDCA may exert additional anti-inflammatory effects by modulating Kupffer cells, the liver-resident macrophages that play a central role in NAFLD pathogenesis, potentially promoting an anti-inflammatory M2-like phenotype and modulating macrophage cytokine production, including TNF-α [32]. UDCA may also modulate the composition of the gut microbiota, and this bile acid–microbiome interaction has been proposed to contribute to its effects in NAFLD [32].

In the 12-week trial by Ji et al. [14], high-sensitivity C-reactive protein (hsCRP) declined modestly in all three groups—placebo, HTD1801 500 mg, and HTD1801 1000 mg (LS mean change: −1.3 [SE 0.3], −1.5 [SE 0.3], and −1.3 [SE 0.3] mg/L, respectively)—with no significant between-group differences (500 mg vs. placebo, *p* = 0.70; 1000 mg vs. placebo, *p* = 0.90). Because hsCRP is a broad and nonspecific marker, these results do not clarify whether HTD1801 meaningfully modulates NLRP3 or NF-κB pathways in humans.

**Figure 3:**
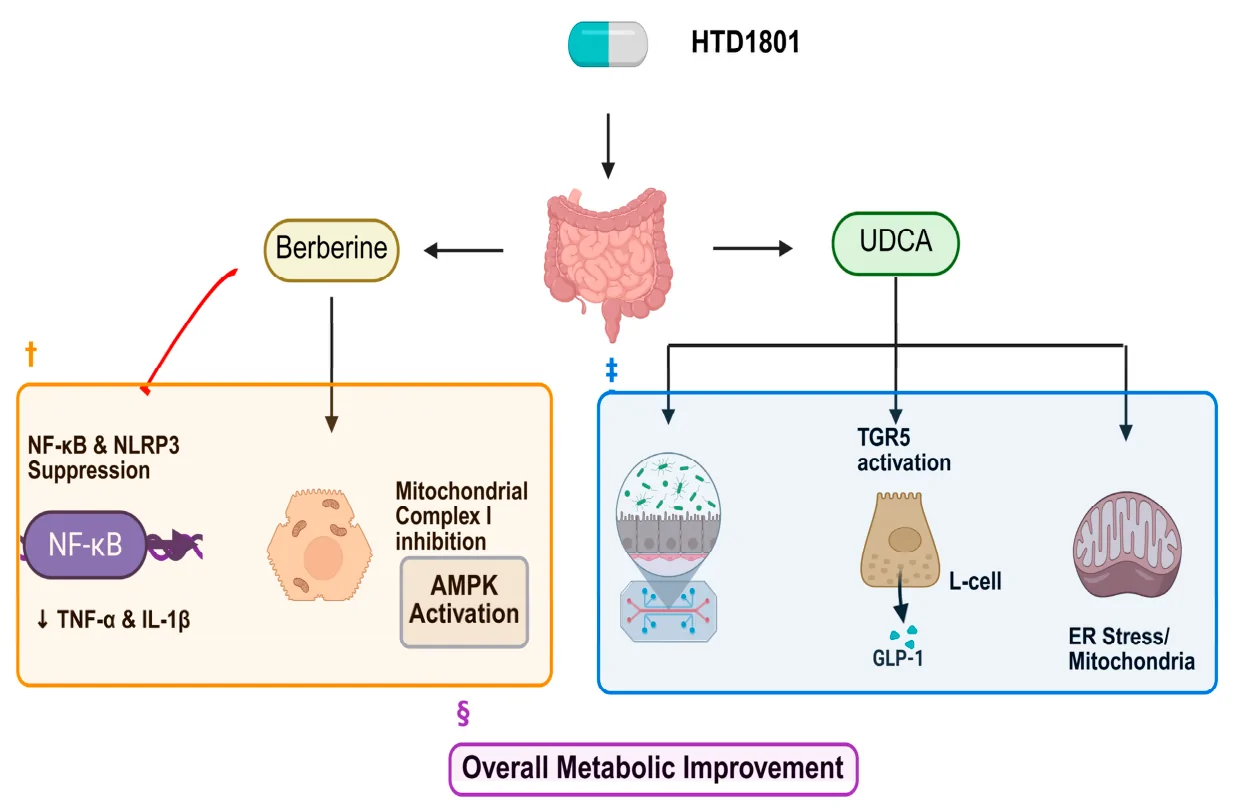
Mechanisms of action of HTD1801. HTD1801 dissociates into berberine and ursodeoxycholic acid (UDCA) in the gastrointestinal tract. † (orange colored box) Berberine (**left**) inhibits mitochondrial complex I, raises the adenosine monophosphate:adenosine triphosphate (AMP:ATP) ratio, and activates AMP-activated protein kinase (AMPK), thereby suppressing hepatic gluconeogenesis and slowing de novo lipogenesis. In preclinical models, berberine also suppresses nuclear factor kappa B (NF-κB) and NOD-, LRR- and pyrin domain-containing protein 3 (NLRP3) signaling, reducing tumor necrosis factor-α (TNF-α), and interleukin-1β (IL-1β). ‡ (blue colored box) UDCA (**right**) may modulate the gut microbiota, activates Takeda G protein-coupled receptor 5 (TGR5) on intestinal L-cells to stimulate endogenous glucagon-like peptide-1 (GLP-1) secretion, and exerts hepatoprotective effects by attenuating endoplasmic reticulum (ER) stress, preventing mitochondrial permeability transition pore (mPTP) opening, and inhibiting Bax-mediated apoptosis. § (purple colored box) These complementary component-level pathways could together contribute to overall metabolic improvement (bottom), including the observed effects on glycemic control, lipid metabolism, inflammation, and hepatic integrity. The proposed mechanisms are derived primarily from studies of berberine and UDCA individually; HTD1801-specific mechanistic evidence is still lacking. Solid arrows indicate activation or direct effects, the red line indicates inhibition, and downward arrows indicate decrease. Created in BioRender. Kim, B. (2026) https://BioRender.com/ycxlxmr (accessed on 30 July 2026).

Another mechanistic thread involves the incretin axis, the gut-hormone system in which intestinally derived hormones such as glucagon-like peptide-1 (GLP-1) are released after nutrient intake to stimulate insulin secretion and enhance glycemic control. UDCA activates the bile acid receptor TGR5 on intestinal L cells (enteroendocrine cells that secrete GLP-1 in response to luminal nutrients), increasing endogenous GLP-1 secretion. Evidence from small human studies suggests this effect: a sequential pre-post study in healthy volunteers by Murakami et al. [33] and a pilot randomized, controlled add-on trial in patients with T2DM and chronic liver disease by Shima et al. [34]. Berberine may also enhance GLP-1 release: in diet-induced obese mice, high-fat feeding caused mitochondrial stress (cristae loss, membrane rupture, and swelling) in colonic enterocytes—the site of GLP-1-producing L cells—and this mitochondrial dysfunction was associated with reduced GLP-1 expression; berberine treatment attenuated the mitochondrial stress and restored GLP-1 expression, an effect attributed to protection of enterocyte mitochondria rather than to changes in gut microbiota [35]. These studies evaluated free UDCA (Murakami [33], Shima [34]) or free berberine (Sun et al. [35]) rather than HTD1801, and no HTD1801 trial has measured endogenous GLP-1 directly. Whether the ionic complex preserves this UDCA-mediated GLP-1 secretory mechanism remains unknown. Overall, the proposed mechanisms of HTD1801, including AMPK activation, attenuation of hepatocellular stress, modulation of inflammatory signaling, and potential enhancement of endogenous GLP-1 secretion, provide a biologically plausible framework for its observed metabolic and hepatic effects (Figure 3). However, current mechanistic understanding remains largely extrapolated from studies of berberine and UDCA individually, as HTD1801-specific mechanistic evidence is still lacking.

## 4. Combination Potential

HTD1801’s broad metabolic and hepatic actions naturally prompt consideration of how it might interact with therapies commonly used in MASLD/MASH and CKM disease. The aim is not to suggest synergy where none has been demonstrated, but to identify areas where biological plausibility and early clinical signals justify further exploration.

### 4.1. HTD1801 Clinical Evidence: An Integrated Summary

Before evaluating potential combination strategies, it is useful to outline what HTD1801 achieves as monotherapy. Across seven completed studies with publicly reported results, from Phase 1b dose escalation work through Phase 3 trials, HTD1801 has shown improvements in several metabolic and liver-related markers including improvements in glycemic control, LDL-C, hepatic fat fraction, and liver enzymes. These effects have appeared across varied study designs, durations, and patient populations, which strengthens confidence in the overall signal.

The phase 1b study evaluated the safety, pharmacokinetics, and pharmacodynamic effects of HTD1801 over 28 days in participants with hypercholesterolemia [19]. At steady state, plasma exposures of both berberine and UDCA appeared broadly dose-proportional across the evaluated dose range. Beyond the pharmacokinetic findings, HTD1801 was generally well tolerated and was associated with dose-dependent reductions in LDL-C and total cholesterol; at the highest dose (2000 mg/day), LDL-C decreased by 10.4% and total cholesterol by 8.2% versus placebo at day 28. However, because the trial did not include a direct comparator group receiving co-administered free berberine and UDCA, it characterizes the pharmacokinetics and short-term pharmacodynamic effects of HTD1801 but does not establish whether the ionic complex confers pharmacokinetic or clinical advantages over its individual components. Although complex formation has been proposed as a strategy to address the poor oral bioavailability of berberine, direct comparative pharmacokinetic and clinical studies are needed.

Two limitations, however, should be acknowledged. First, the completed Phase 3 T2DM trials have been conducted in China [15,16,17], while the earlier MASH/T2DM Phase 2 trial was conducted in the United States [13], and the Phase 1b/2a mechanistic and safety studies were conducted in Australia [19] and China [18]. This does not diminish the value of the findings, but metabolic disease varies across ethnic backgrounds, and broader geographic validation will be essential. Second, no published human study has yet reported biopsy-confirmed histologic efficacy outcomes for HTD1801. The CENTRICITY Phase 2b trial (NCT05623189) was completed with biopsy-based histologic endpoints, but results have not been posted on ClinicalTrials.gov or published as of 9 August 2026 [36]. A 2025 secondary analysis combined preclinical histology with human noninvasive markers rather than human biopsy outcomes [37], and subsequent commentary highlighted the limits of inferring histologic benefit from surrogate markers [38]. Improvements in ALT, AST, or MRI-PDFF remain encouraging but cannot substitute for direct histologic confirmation of MASH resolution or fibrosis improvement. Until such data are available, HTD1801’s position among liver-directed therapies will remain provisional.

These considerations help clarify where HTD1801 currently stands and what questions remain open as its potential role alongside other agents is evaluated.

### 4.2. Metformin

**Figure 4:**
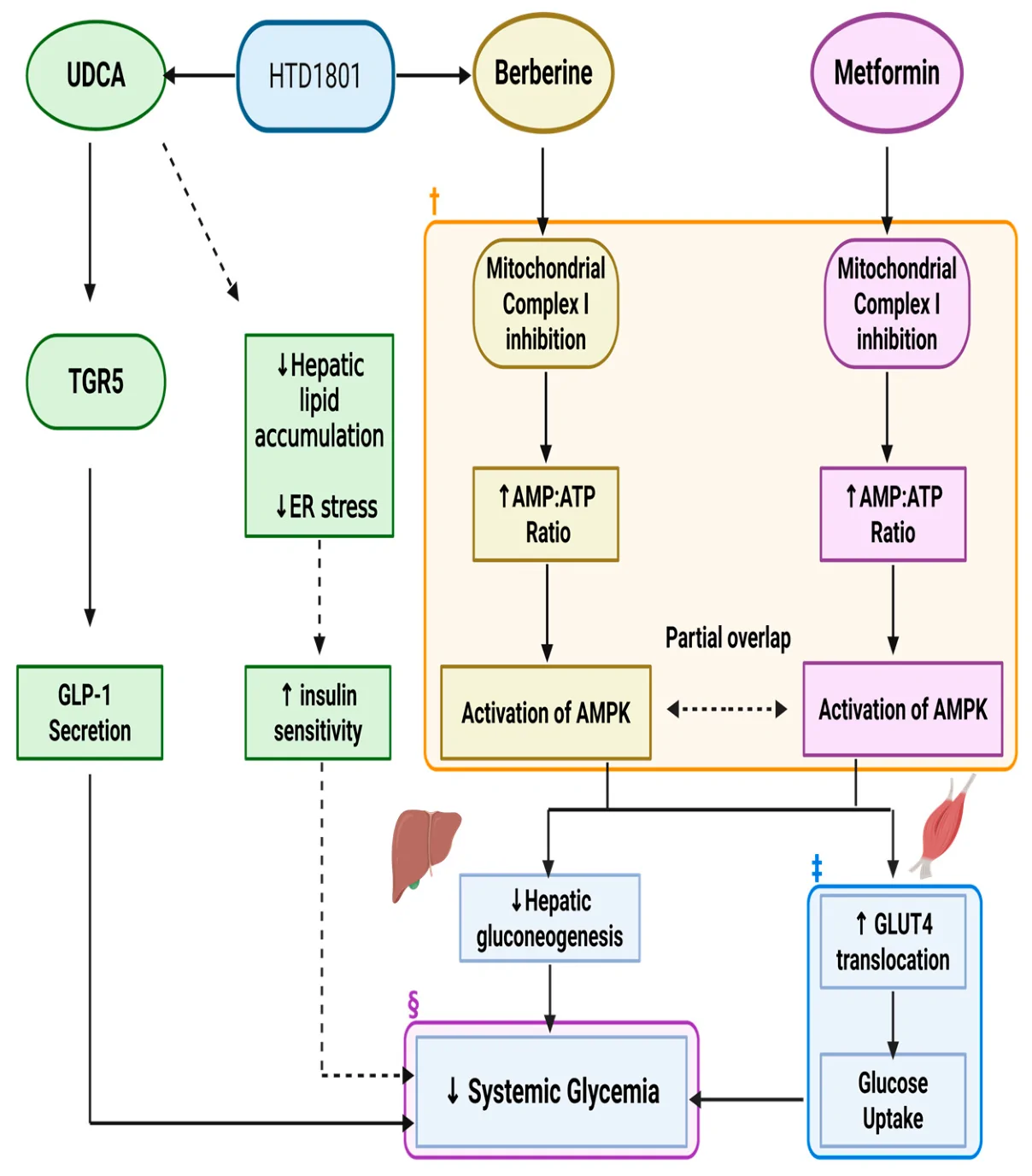
Mechanisticrationale for combining HTD1801 with metformin. † (orange colored box) Both berberine and metformin inhibit mitochondrial complex I, elevate the adenosine monophosphate:adenosinetriphosphate (AMP:ATP) ratio, and activate AMP-activated protein kinase (AMPK), resulting in reduced hepatic gluconeogenesis (partial mechanistic overlap). ‡ (blue colored box) Both agents enhance peripheral glucose uptake via AMPK-mediated glucose transporter type 4 (GLUT4) translocation in skeletal muscle and adipose tissue. § (purple colored box) The effects of UDCA and the AMPK-mediated hepatic and peripheral effects converge on reduced systemic glycemia. Ursodeoxycholic acid (UDCA) complements these actions through Takeda G protein-coupled receptor 5 (TGR5)-mediated glucagon-like peptide-1 (GLP-1) secretion and direct hepatoprotection (reduced endoplasmic reticulum stress and lipid accumulation), and may improve hepatic insulin sensitivity. Solid arrows indicate activation or direct effects, dashed arrows indicate indirect effects or partial overlap, and upward/downward arrows indicate increase/decrease. Created in BioRender. Kim, B. (2026) https://BioRender.com/9lffxwl (accessed on 30 July 2026).

Both metformin and berberine inhibit mitochondrial complex I, raise the AMP:ATP ratio, and activate AMPK, thereby suppressing hepatic gluconeogenesis [25,39,40]. This shared mechanism produces partial overlap. Berberine monotherapy has also been shown to increase glucose disposal rate on hyperinsulinemic-euglycemic clamp testing, consistent with improved insulin sensitivity [41]. In addition, both agents enhance peripheral glucose uptake via AMPK-mediated GLUT4 translocation [42]. UDCA may complement these effects by stimulating TGR5-dependent GLP-1 secretion and exerting direct hepatoprotective actions; in KK-Ay mice (a genetic mouse model of obesity-associated type 2 diabetes), UDCA improved hepatic insulin sensitivity without affecting peripheral parameters [43]. Because these findings derive from studies of the individual components rather than the HTD1801 complex, they provide only indirect support for combination therapy (Figure 4).

In high-fat diet mouse models and HepG2 hepatocyte cultures, co-administration of berberine and metformin yielded greater reductions in steatosis, inflammation, and hepatocyte ballooning than either agent alone through an AMPK-dependent pathway [44]. Although these combination studies did not include UDCA, and therefore inform HTD1801 pharmacology only indirectly, they provide supportive mechanistic rationale for targeting complementary metabolic pathways.

Clinical evidence supporting this rationale comes from the Phase 3 SYMPHONY-2 trial, which evaluated HTD1801 as add-on therapy in patients with type 2 diabetes inadequately controlled on metformin [16]. Over 24 weeks, add-on HTD1801 achieved a significantly greater reduction in HbA1c compared with placebo (adjusted least-squares mean difference, −0.5%; 95% CI, −0.7 to −0.4; *p* < 0.0001). A greater proportion of patients receiving HTD1801 achieved HbA1c < 7.0% (33% vs. 11%), accompanied by improvements in LDL-C and GGT. SYMPHONY-2 was designed to assess overall therapeutic efficacy rather than to isolate individual mechanistic contributions.

Among the combination strategies evaluated to date, HTD1801 plus metformin has the most mature clinical evidence, supported by Phase 3 randomized trial data. This combination may be particularly relevant for patients with T2DM inadequately controlled on metformin, especially those with concurrent MASLD, dyslipidemia, or elevated liver enzymes. Beyond glycemic improvement, the potential hepatic and metabolic benefits of HTD1801 make this combination an attractive area for further investigation in high-risk metabolic populations [16].

Recent studies have identified additional mechanisms for metformin beyond AMPK activation. Gut-derived GDF15 release has been identified as a major contributor to the acute metformin-induced rise in circulating GDF15 [45]. In addition, in mice with diet-induced metabolic syndrome, the benefits of metformin on obesity, hepatic steatosis, and low-grade inflammation persisted after antibiotic depletion of the microbiota and in germ-free animals, indicating anti-inflammatory activity that does not depend on the gut microbiota [46]. Both pathways are independent of berberine and UDCA, derive mainly from preclinical work, and have not been linked to the clinical benefits seen in SYMPHONY-2. Even so, they broaden the mechanistic rationale for metformin as a metabolic therapy.

Physical activity is a cornerstone of MASLD and T2DM management and may interact with insulin-sensitizing agents. In a randomized, placebo-controlled trial of metformin-naïve adults with metabolic syndrome (*n* = 24 randomized; 22 completed), five weeks of aerobic training combined with metformin produced greater fat oxidation but a smaller gain in VO_2_peak than training with placebo [47]; attenuation of training-induced improvements in cardiorespiratory fitness and insulin sensitivity has also been reported in older adults [48]. Because berberine, like metformin, inhibits mitochondrial complex I [25], a comparable interaction with HTD1801 is conceivable but has not been studied and remains hypothetical.

### 4.3. GLP-1 Receptor Agonists

**Figure 5:**
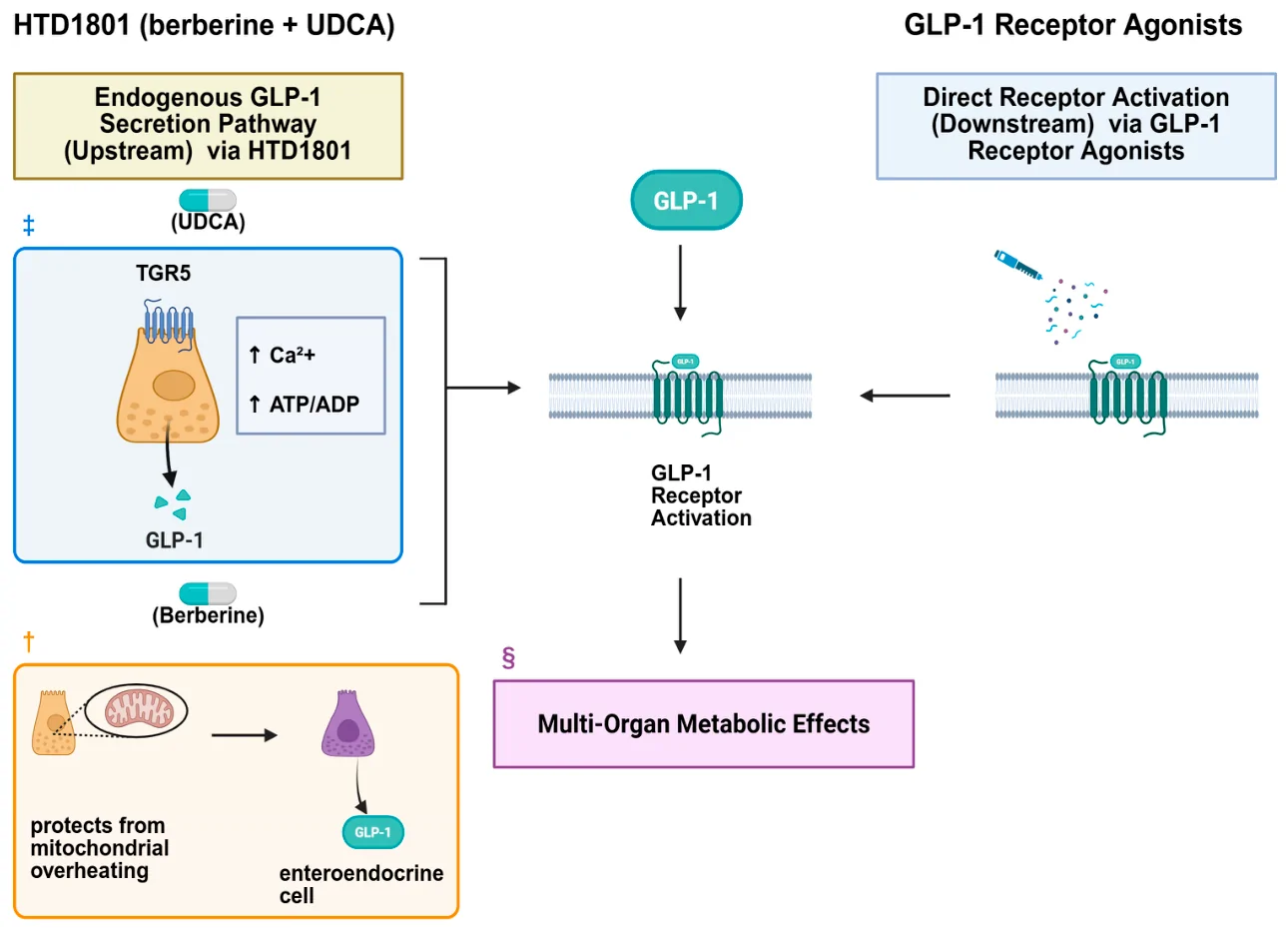
Proposed complementary mechanisms of HTD1801 and glucagon-like peptide-1 (GLP-1) receptor agonists. ‡ (blue colored box) Ursodeoxycholic acid (UDCA) stimulates endogenous GLP-1 secretion via Takeda G protein-coupled receptor 5 (TGR5) activation on intestinal L-cells, increasing intracellular Ca^2+^ and the ATP/ADP ratio. † (orange colored box) Berberine may further support GLP-1 secretion by protecting colonic enterocytes from mitochondrial overheating (**bottom left**). Together, ‡ (blue colored box) and † (orange colored box) represent the upstream, endogenous GLP-1 secretion pathway of HTD1801. In contrast, GLP-1 receptor agonists (**top right**) directly activate the GLP-1 receptor. § (purple colored box) Both routes converge on GLP-1 receptor activation, resulting in multi-organ metabolic effects. Because HTD1801 acts upstream to increase endogenous ligand availability while GLP-1 receptor agonists act downstream at the receptor, the two approaches are potentially complementary rather than fully redundant. Acting at different points along the GLP-1 pathway does not by itself establish additive or synergistic clinical benefit, however; this remains a hypothesis to be tested in dedicated combination trials, and clinical evidence for the combination remains limited. Created in BioRender. Kim, B. (2026) https://BioRender.com/7zxywys (accessed on 30 July 2026).

GLP-1, released from intestinal L-cells, lowers blood glucose by stimulating insulin secretion and suppressing glucagon in a glucose-dependent manner, while also delaying gastric emptying and reducing appetite [49,50]. GLP-1 receptor agonists continuously activate the GLP-1 receptor independently of endogenous hormone levels. By comparison, the components of HTD1801 act further upstream: UDCA stimulates endogenous GLP-1 secretion through TGR5 activation on L-cells, whereas berberine has been shown in diet-induced obese mice to restore GLP-1 secretion by protecting colonic enterocytes from mitochondrial overheating [33,34,35]. Because HTD1801 enhances endogenous GLP-1 availability whereas GLP-1 receptor agonists directly activate the receptor, these therapies act at different levels of the GLP-1 pathway and may therefore provide complementary therapeutic effects rather than redundant ones (Figure 5).

Support for this combination comes primarily from translational animal models. In a diet-induced obesity mouse study presented at the 2026 ADA Scientific Sessions, combining HTD1801 with semaglutide or tirzepatide produced greater weight loss than either GLP-1 receptor agonist alone, primarily through additional reductions in fat mass while largely preserving lean mass [51]. Following semaglutide discontinuation, HTD1801 also attenuated body-weight regain, improved body composition by maintaining a lower fat-mass ratio and a higher lean-mass ratio, and reduced glucose rebound. Although these findings are currently available only in a conference abstract and await peer-reviewed quantitative confirmation, they suggest a potential role for HTD1801 in supporting weight maintenance and preserving favorable body composition.

Dual and triple incretin-based agonists, such as tirzepatide and retatrutide, produce substantial weight loss and liver-fat reduction. In the phase 2 SYNERGY-NASH trial, tirzepatide increased the proportion of participants with biopsy-assessed MASH resolution without worsening of fibrosis compared with placebo [52], whereas the phase 2a retatrutide study reported large reductions in MRI-PDFF liver fat without histologic assessment [53]. Histologic improvement has not yet been demonstrated for HTD1801, and no head-to-head comparison exists. HTD1801 may therefore be better positioned as an adjunctive therapy than as a competitor, potentially for patients who show an incomplete response to these agents.

Beyond obesity and MASLD, polyendocrine metabolic ovarian syndrome (PMOS) is an additional insulin-resistant phenotype relevant to the CKM continuum. Insulin resistance is recognized as a key feature of PMOS, and metabolic risk factors, diabetes, and cardiovascular disease are increased in affected women [54]. Because insulin resistance is central to the condition, insulin-sensitizing mechanisms such as AMPK activation by berberine [25] and metformin [39] are of interest. In a 2026 systematic review and meta-analysis of randomized trials, GLP-1 receptor agonists were associated with modest short-term weight loss in women with PMOSand overweight or obesity, whereas evidence for benefit on other metabolic and reproductive outcomes remained of low certainty [55]. Berberine, one of the two components of HTD1801, has also been evaluated for PMOS-associated insulin resistance; a meta-analysis of nine randomized trials found no significant difference between berberine and metformin for insulin resistance or glycolipid metabolism, although the authors judged the available data insufficient for firm conclusions [56]. No HTD1801-specific data exist in this population. PMOS thereforeillustrates the potential breadth of the CKM framework beyond MASLD/MASH and cardiovascular disease, but it remains a direction for future investigation rather than an established combination indication.

HTD1801 may represent a rational combination partner for patients with obesity-associated MASLD who continue to exhibit hepatic steatosis or dyslipidemia despite GLP-1 receptor agonist-induced weight loss. GLP-1 receptor agonists improve hepatic outcomes primarily through systemic weight reduction, whereas UDCA directly protects hepatocytes by attenuating endoplasmic reticulum stress, preventing mitochondrial permeability transition pore opening, and inhibiting Bax-mediated apoptosis (Figure 3) [29]. These mechanistic differences raise the possibility that HTD1801 may help address persistent hepatocellular injury independently of weight loss. This hypothesis warrants evaluation in early-phase clinical combination studies.

### 4.4. Statins

**Figure 6:**
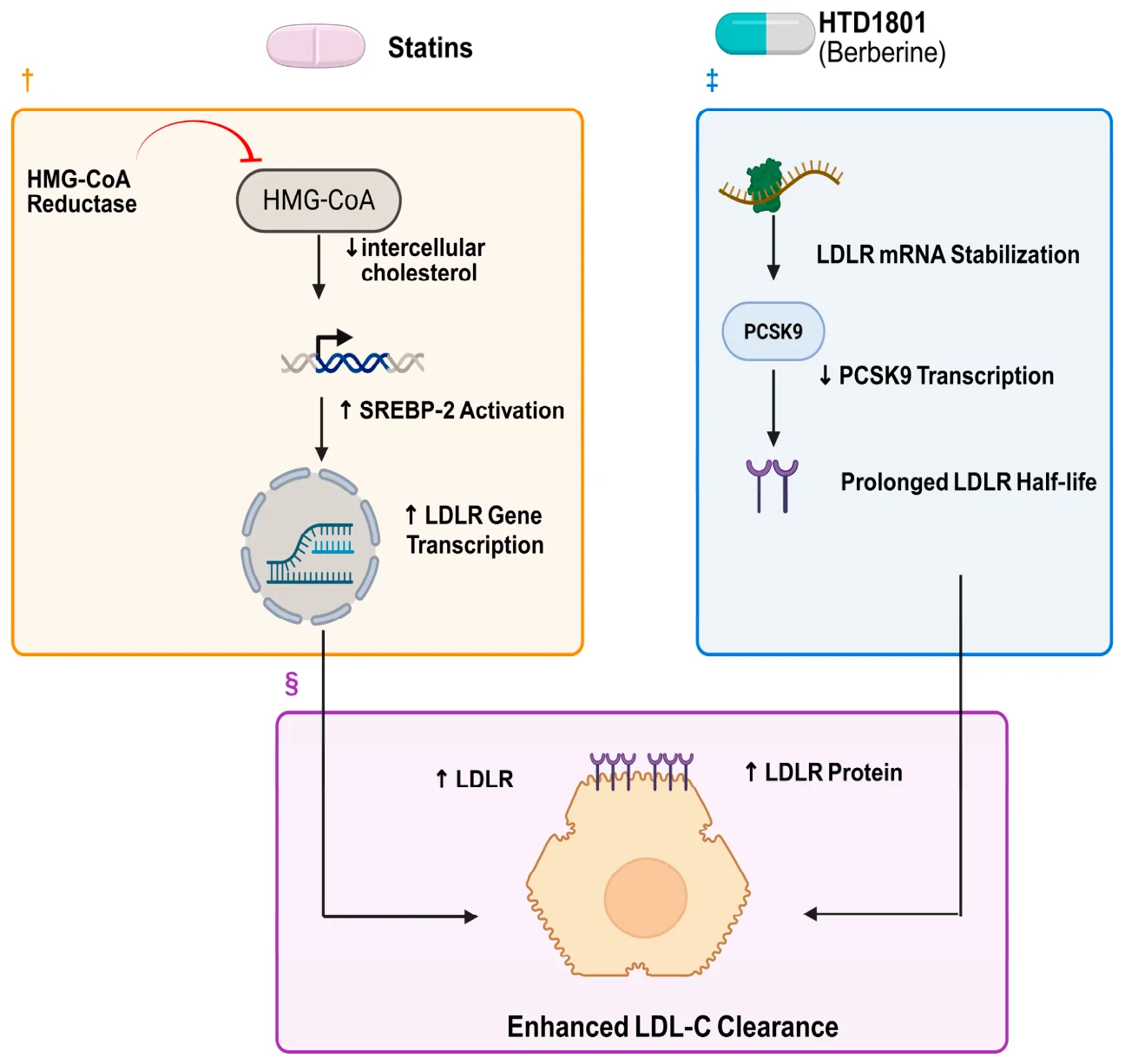
Mechanistic rationale for combining HTD1801 with statins. † (orange colored box) Statins inhibit 3-hydroxy-3-methylglutaryl-coenzyme A (HMG-CoA) reductase, lowering intracellular cholesterol, which activates sterol regulatory element-binding protein 2 (SREBP-2) and increases low-density lipoprotein receptor (LDLR) gene transcription (left). ‡ (blue colored box) The berberine component of HTD1801 stabilizes LDLR mRNA and suppresses proprotein convertase subtilisin/kexin type 9 (PCSK9) transcription, thereby prolonging LDLR half-life (right). § (purple colored box) Through these mechanistically distinct pathways, both agents increase LDLR protein on the hepatocyte surface and enhance low-density lipoprotein cholesterol (LDL-C) clearance, supporting a mechanistic rationale for prospective evaluation of statins with HTD1801. Solid arrows indicate activation or promotion, the red line indicates inhibition, and upward/downward arrows indicate increase/decrease. Created in BioRender. Kim, B. (2026) https://BioRender.com/cue2em9 (accessed on 30 July 2026).

Statins remain the cornerstone of LDL-C-lowering therapy, reducing cardiovascular risk primarily by inhibiting HMG-CoA reductase and upregulating LDL receptor transcription through SREBP-2 [57,58]. Despite their proven efficacy, many patients do not achieve guideline-recommended LDL-C targets with statin monotherapy, leaving substantial residual cardiovascular risk [59]. Berberine offers a distinct complementary mechanism by stabilizing LDLR mRNA and suppressing PCSK9 transcription [60,61,62,63]. Because statin therapy also induces a compensatory increase in circulating PCSK9 levels, partially limiting its LDL-C-lowering effect, berberine-mediated PCSK9 suppression may counteract this adaptive response [64] (Figure 6). These complementary mechanisms support further clinical evaluation of HTD1801 in combination with statins to improve lipid control.

In high-fat diet-fed rats, co-administration of berberine (90 mg/kg/day) and simvastatin (6 mg/kg/day) reduced serum LDL-C by 46.2%, exceeding the reductions achieved with simvastatin (28.3%) or berberine (26.8%) alone (*p* < 0.01 for both). This combined effect was comparable to high-dose simvastatin monotherapy (12 mg/kg/day; 43.4%) and was accompanied by an approximately 1.6-fold increase in hepatic LDLR mRNA expression [65].

In monocyte-derived macrophages, berberine (0.1–100nM) combined with atorvastatin (100 nM) dose-dependently suppressed LOX-1 expression through an endothelin-1 receptor-mediated mechanism [66]. Although these studies evaluated berberine rather than HTD1801 directly, Phase 2 clinical trials have demonstrated that HTD1801 independently lowers LDL-C, providing indirect clinical support for further evaluation of this combination strategy [13,14].

HTD1801 may represent a potential add-on option for patients with MASLD or T2DM who continue to exhibit atherogenic dyslipidemia despite optimized statin therapy. The complementary mechanisms of statin-mediated cholesterol synthesis inhibition and berberine-mediated stabilization of hepatic LDL receptors provide a biological rationale for combination therapy. Whether this mechanistic complementarity translates into additional clinical benefit remains to be established in dedicated prospective combination trials [59].

### 4.5. SGLT2 Inhibitors

SGLT2 inhibitors lower blood glucose through glucosuria and confer robust cardiorenal protection via mechanisms distinct from those of HTD1801 [9,67,68,69]. Although certain downstream pathways, such as AMPK activation and NLRP3 inflammasome suppression, may partially overlap with the pharmacological actions of berberine or ursodeoxycholic acid (UDCA), the two therapies primarily act through distinct physiological mechanisms, and evidence to date is comparative (head-to-head) rather than combination-based.

In addition to individual clinical studies, several meta-analyses of randomized controlled trials have reported consistent hepatic benefits of SGLT2 inhibitors that further support their potential role in this therapeutic setting. In patients with T2DM and NAFLD/MASLD, pooled analyses have shown significant reductions in alanine aminotransferase (weighted mean difference [WMD], −5.36 U/L; 95% CI, −8.86 to −1.85) and hepatic fat fraction (WMD, −2.20%; 95% CI, −3.67 to −0.74) [70], with a more recent meta-analysis of MRI-based studies confirming a moderate-to-large reduction in liver fat content (standardized mean difference, −0.73; 95% CI, −0.97 to −0.50) [71]. A separate meta-analysis focusing on non-invasive fibrosis indices further reported modest improvements in the NAFLD fibrosis score and, at borderline significance, the Fibrosis-4 index (*p* = 0.05), although liver stiffness measurements did not differ significantly from control [72]. In women with PMOS, two meta-analyses of randomized trials have examined SGLT2 inhibitors. An earlier analysis of four trials (158 participants) reported improvements in body weight, fasting glucose, and HOMA-IR [73], whereas a more recent analysis of five trials (205 participants), mostly against active comparators, confirmed a reduction in HOMA-IR and reductions in total testosterone and free androgen index. However, this analysis found no significant change in body weight, small increases in total and LDL cholesterol, and more genitourinary adverse events, and trial sequential analysis indicated that the evidence was underpowered [74]. Collectively, this meta-analytic evidence, together with the head-to-head HARMONY findings described below, strengthens, though does not yet establish, the rationale for evaluating HTD1801 in combination with SGLT2 inhibitors, pending peer-reviewed publication of the underlying individual trials.

Although combination therapy with SGLT2 inhibitors has not yet been formally evaluated, head-to-head clinical data provide indirect evidence relevant to future combination strategies. The Phase 3 HARMONY trial (NCT06415773) compared HTD1801 with dapagliflozin head-to-head as add-on therapy in patients with T2DM inadequately controlled on background metformin, with both arms continuing metformin † [17]. The trial was designed to test non-inferiority of HTD1801 against dapagliflozin for HbA1c reduction, using a pre-specified margin of 0.4%. At week 24, HTD1801 met this non-inferiority criterion. Sponsor-reported and conference-presented data further indicated a numerically greater within-group HbA1c reduction with HTD1801 than with dapagliflozin (−1.12% vs. −0.93%) and a higher proportion of patients achieving HbA1c < 7.0%. The exploratory between-group least-squares mean difference was −0.20% (95% CI, −0.37 to −0.03; reported *p* < 0.001, not yet independently verified). HTD1801 treatment was also associated with a within-group increase in eGFR during the study period, whereas dapagliflozin did not demonstrate a comparable increase; a formal between-group comparison of eGFR has not been reported. Serious adverse events occurred in 3.8% of HTD1801-treated patients versus 4.4% with dapagliflozin. These findings remain preliminary, as HARMONY has so far been reported only in conference and press-release form, until full peer-reviewed results are available.

Patients with cardiovascular–kidney–metabolic (CKM) syndrome, particularly those with concomitant MASLD and early chronic kidney disease, may represent an appropriate population for future combination studies. The established cardiorenal benefits of SGLT2 inhibitors and the hepatic and glycemic effects of HTD1801 suggest complementary therapeutic roles. Whether these complementary mechanisms translate into additional clinical benefit should be evaluated in prospective combination trials [5,75].

### 4.6. Low-Evidence Candidates: PPAR Agonists and Resmetirom

PPAR agonists and resmetirom act through mechanisms distinct from the AMPK- and TGR5-mediated actions of HTD1801. PPAR agonists improve insulin sensitivity while attenuating inflammatory and fibrotic pathways, whereas resmetirom selectively enhances mitochondrial fattyacid oxidation through thyroid hormone receptor-β (THRβ) activation [76]. Clinically, lanifibranor demonstrated histologic efficacy in the Phase 2b NATIVE trial [77], and resmetirom achieved biopsy-confirmed histologic improvements in pivotal Phase 3 studies [12]; evidence relevant to HTD1801 combination with either agent, however, remains theoretical.

Although these distinct mechanisms provide a theoretical rationale for combination therapy, no preclinical or clinical studies have evaluated HTD1801 in combination with either PPAR agonists or resmetirom. While HTD1801 consistently improves hepatic fat fraction, liver transaminases, and systemic lipid profiles, its clinical positioning relative to histology-proven therapies such as resmetirom or pan-PPAR agonists remains uncertain because published human biopsy-confirmed histologic efficacy data are not yet available. Among the drug classes reviewed, evidence supporting combination therapy with PPAR agonists or resmetirom remains limited and largely theoretical.

## 5. Discussion

Current evidence provides several rationales for combining HTD1801 with established therapies, yet the strength of clinical support varies widely across drug classes. Metformin is the only partner backed by Phase 3 randomized data demonstrating the efficacy of HTD1801 as add-on therapy (SYMPHONY-2) [16]. GLP-1 receptor agonists and statins act through complementary pathways that make combination therapy biologically plausible [33,60,62], but no clinical combination studies have been conducted. For SGLT2 inhibitors, the available data come mainly from head-to-head comparisons, while evidence for PPAR agonists and resmetirom is still largely mechanistic [11,12]. In short, mechanistic insights have advanced faster than clinical validation. The documented ability of UDCA to stimulate GLP-1 via TGR5 [33,34] and the consistent LDL-C reductions seen with HTD1801 monotherapy [13,14] offer a reasonable foundation for testing combinations with GLP-1 receptor agonists and statins in future trials.

HTD1801 improves glycemic control and lipid profiles and produces favorable trends in hepatic and renal biomarkers, which supports its further evaluation in the context of CKM syndrome [5,78]. Several important limitations, however, must be kept in mind. No published human MASH trial has reported biopsy-confirmed histologic efficacy results for HTD1801, and direct comparisons with other liver-directed agents are lacking. In addition, geographic representation remains limited: the Phase 3 T2DM studies to date have been conducted in China [15,16], the earlier MASH/T2DM Phase 2 trial was conducted in the United States [13], and the earlier Phase 1b/2a mechanistic and safety studies were conducted in Australia [19]. Additionally, much of the mechanistic understanding still rests on studies of berberine or UDCA alone rather than the ionic complex itself [25,29]. A substantial portion of the clinical data also comes from sponsor-affiliated reports or conference abstracts that have not yet undergone full peer review. Interactions with physical activity were examined only for metformin; other drug classes were not systematically reviewed, and future HTD1801 trials should record or stratify by exercise.

Beyond its effects on glucose, HTD1801 has been linked to improvements in several cardiometabolic markers: reductions in hepatic fat (−4.8% on MRI-PDFF), ALT, AST, LDL-C, and GGT, as well as increases in eGFR, particularly in patients with mild chronic kidney disease [13,14,15,16,17]. A pooled posthoc analysis of SYMPHONY-1 and SYMPHONY-2 presented at the 2026 ERA Congress showed that patients with baseline eGFR of 60–90 mL/min/1.73 m^2^ had a mean eGFR increase of +3.08 mL/min/1.73 m^2^ at week 52 (95% CI 0.46–5.70), without signs of hyperfiltration or fluid retention [79]. Taken together, these observations raise the possibility that HTD1801 could help address residual risks such as dyslipidemia, hepatic steatosis, and early renal impairment even after glycemic targets are met. Because the trials were not designed or powered to assess hard cardiometabolic outcomes; however, this interpretation remains exploratory [13,14].

Both SYMPHONY trials stratified randomization by a baseline HbA1c threshold of 8.5%, yet peer-reviewed subgroup analyses examining differential treatment response by glycemic severity have not been published. It remains uncertain whether HTD1801 is best positioned as add-on therapy for patients inadequately controlled on metformin or whether it may also have a role earlier in treatment. Prospective studies with predefined HbA1c strata will be needed to clarify this question.

Patients with MASLD and T2DM frequently exhibit overlapping features of CKM syndrome, making it unlikely that any single pathway agent can fully address the combined metabolic, hepatic, cardiovascular, and renal abnormalities [5]. This context supports further evaluation of HTD1801 in combination with agents such as GLP-1 receptor agonists [80,81], SGLT2 inhibitors, and resmetirom. In March 2026, China’s National Medical Products Administration accepted HighTide Therapeutics’ first New Drug Application for HTD1801, based on three Phase 3 trials that met their primary and multiple secondary endpoints in T2DM, a notable regulatory milestone toward potential commercialization [82].

Based on the evidence-gap analysis in Table 2, the highest-priority next steps are as follows: (1) publication of the pending biopsy-based histologic results from the completed CENTRICITY Phase 2b trial (NCT05623189); (2) a dedicated combination RCT with GLP-1 receptor agonists, given the strongest mechanistic rationale among untested partners; and (3) confirmatory Phase 3 data in populations outside the completed Chinese cohorts. Based on current evidence, HTD1801 may represent a reasonable candidate for add-on therapy in patients who continue to carry residual cardiometabolic risk despite metformin, although this positioning will require confirmation in adequately powered trials.

As HTD1801 remains investigational, no drug-acquisition price currently exists. Estimating a total treatment cost would therefore be premature. Even so, several cost drivers can already be anticipated. These include the eventual drug price, monitoring costs such as liver enzymes, lipid panels, and HbA1c, and the duration of treatment needed to sustain benefit, since durability beyond the 24–52-week trial windows reported to date remains unknown. We recommend that future Phase 3 and combination trials collect resource-utilization data alongside efficacy endpoints. This would allow a formal cost-effectiveness analysis once pricing is established, benchmarked against metformin-, incretin-, and SGLT2 inhibitor-based strategies in the healthcare systems where HTD1801 may eventually be marketed—initially China, where a New Drug Application has been accepted for review [82], and potentially the United States. The incremental cost-effectiveness of an HTD1801 add-on will depend heavily on the comparator strategy, which ranges from generic metformin to branded incretin therapies, and on the health system considered.

Combination strategies are likely to be most effective when guided by patient phenotype rather than drug class alone. Individuals with obesity-associated MASLD may be particularly suitable for evaluating HTD1801 together with GLP-1 receptor agonists [83,84], whereas patients with persistent dyslipidemia despite statin therapy [59] or those with CKM syndrome and early CKD [5,75] may be better candidates for statin or SGLT2 inhibitor-based combinations. At present, metformin remains the only partner supported by Phase 3 randomized clinical evidence for HTD1801 add-on therapy [16]. Although these concepts are still exploratory, a phenotype-guided approach may improve both trial design and patient selection in future combination studies. Table 2 summarizes the current mechanistic, preclinical, and clinical evidence for each candidate partner.

## 6. Safety Considerations

Before considering combination-specific risks, it is worth noting the baseline toxicity profile of HTD1801’s components. Berberine is generally well tolerated, with gastrointestinal upset (abdominal pain, diarrhea, constipation) as the most common adverse effect; rarer but clinically significant risks include dose-dependent cardiotoxicity and hemolytic jaundice/kernicterus in G6PD-deficient neonates, historically leading to contraindication in pregnancy and infancy [85,86]. UDCA is comparatively well tolerated, with dose-related diarrhea as its main adverse effect [23]. These baseline risks are relevant to combination potential because they define which patient populations (e.g., pregnant patients, those with pre-existing bradyarrhythmia) and which co-administered drug classes (e.g., other QT-prolonging or CYP3A4-dependent narrow-therapeutic-index agents) warrant particular caution. No HTD1801-specific overdose data have been published.

When evaluating HTD1801 in combination with other therapies, the main safety considerations involve overlapping adverse effects and potential pharmacokinetic interactions. Observed clinical adverse events: in SYMPHONY-2, diarrhea was the most frequently reported adverse event with HTD1801 add-on therapy (23.8% vs. 1.1% with placebo, including three severe cases) [16]; treatment discontinuation and serious adverse event rates specific to SYMPHONY-2 were not separately highlighted in the available report and should be confirmed against the primary publication. In the head-to-head HARMONY trial, serious adverse events occurred in 3.8% of HTD1801-treated patients versus 4.4% with dapagliflozin, with no severe hypoglycemia in the HTD1801 arm [17]. Because metformin commonly causes gastrointestinal intolerance, this overlap is particularly relevant for the combination with the strongest supporting evidence. Even so, no unexpected safety signals emerged in that trial [16].

Theoretical, combination-specific tolerability concerns: UDCA can stimulate endogenous GLP-1 secretion through TGR5 activation, raising the possibility of increased gastrointestinal intolerance when HTD1801 is paired with GLP-1 receptor agonists; however, this combination has not yet been evaluated clinically [33,87,88]. Resmetirom has shown an acceptable safety profile in Phase 3 studies [12], but its concomitant use with HTD1801 remains unexplored. PPAR agonists such as lanifibranor are associated with diarrhea, nausea, peripheral edema, anemia, and weight gain [77], making gastrointestinal tolerability a potential concern if used together with HTD1801.

Whether these adverse effects interact with HTD1801’s metabolic or hepatic actions is unknown. SGLT2 inhibitors promote osmotic diuresis and increase the risk of genital infections; although their mechanisms differ from those of HTD1801, combined therapy may require monitoring of volume status and renal function in susceptible individuals [89].

Theoretical, mechanism-based interaction concerns (not yet confirmed for HTD1801 itself): Repeated berberine administration has been reported to inhibit CYP3A4 and CYP2D6 activity in humans [90], and preclinical work has raised concern about combined CYP3A4 inhibition and hERG channel effects when berberine is used with selected statins [91]. This creates a theoretical interaction concern for statins that depend substantially on CYP3A4 metabolism, including simvastatin and atorvastatin [92]. However, these findings have not been established specifically for HTD1801 in prospective drug–drug interaction studies. Direct HTD1801 interaction data are needed before preferential statin selection, routine ECG monitoring, or other combination-specific monitoring strategies can be recommended. Clinically meaningful CYP-mediated interactions are less expected with metformin, GLP-1 receptor agonists, or SGLT2 inhibitors, but dedicated HTD1801 studies remain limited.

An additional, HTD1801-specific consideration is that berberine’s systemic exposure depends heavily on gut-microbiota-mediated conversion to its more absorbable form, dihydroberberine [93], while UDCA undergoes its own microbiota-dependent processing [94]. Because both components share the same luminal microbial community, agents that alter gut microbiota composition—including metformin and other combination partners discussed in this review—could in principle modify berberine’s absorption and, consequently, the magnitude of its CYP-mediated interactions; this has not been directly tested for HTD1801.

Gradual titration of incretin-based therapies may help improve gastrointestinal tolerability. Routine monitoring of liver enzymes and lipid profiles is appropriate during treatment, and MRI-PDFF, when available, may offer additional insight into hepatic response [6]. Completed clinical studies have not identified unexpected safety concerns with HTD1801, but combination-specific safety data remain limited. Future trials designed to evaluate combination therapy should incorporate dedicated safety assessments.

## 7. Conclusions

HTD1801 engages multiple pathways involved in metabolic dysfunction, hepatic injury, and dyslipidemia. Across completed clinical studies, it has consistently improved glycemic control, LDL-C, hepatic fat content, and liver enzyme profiles. These findings support continued evaluation of HTD1801 as an adjunctive therapy for patients with MASLD/MASH or CKM syndrome who continue to carry residual cardiometabolic risk despite standard treatment.

Several evidence gaps still shape its current positioning. Published human biopsy-based histologic efficacy has not yet been demonstrated, all completed Phase 3 T2DM trials have been conducted exclusively in Chinese cohorts, and no dedicated human combination studies have assessed HTD1801 with GLP-1 receptor agonists, statins, resmetirom, or PPAR agonists. In addition, although HTD1801 was developed as an ionic complex of berberine and UDCA, it remains uncertain whether the complex provides pharmacokinetic or clinical advantages beyond co-administration of the two components because direct comparative studies are lacking.

The role HTD1801 ultimately occupies in MASH and CKM care will depend on publication of the biopsy-based outcomes from the completed CENTRICITY trial and on confirmation of efficacy and safety in more diverse populations. Favorable results would strengthen the case for incorporating HTD1801 into phenotype-guided combination strategies within the CKM framework.

## Figures and Tables

**Table 1:** Summary of completed clinical trials of HTD1801: study designs, endpoints, and key findings. Theoverall pattern is clear: HTD1801 reliably improves metabolic and hepatic markers, and several studies demonstrate dose-responsive effects.

Trial ID/Reference/CEBM Evidence Level	Population/Design	N/Duration; Nation	Primary Endpoint	Key Results	Notable Secondary Findings
NCT03656744 (Phase 2a) Harrison et al. [13] Level 1b	MASH + T2DM; 1:1:1 double-blind (DB): HTD1801 500 mg BID (1000 mg/day) vs. 1000 mg BID (2000 mg/day) vs. placebo (monotherapy)	N = 100; 18 weeks; USA	MRI-PDFF (magnetic resonance imaging-proton density fat fraction) hepatic fat reduction	1000 mg: −4.8% vs. placebo −2.0% (*p* = 0.011); MRI response 52% vs. 24%	ALT/AST improvement; LDL-C reduction; HbA1c improvement; dose-dependent HOMA-IR reduction
NCT06411275 (Phase 2) Ji et al. [14] Level 1b	T2DM inadequately controlled with diet/exercise; 1:1:1 DB RCT: HTD1801 500 mg BID (1000 mg/day) vs. 1000 mg BID (2000 mg/day) vs. placebo (monotherapy)	N = 113; 12 weeks; China	HbA1c reduction	500 mg: −0.4% vs. placebo (*p* = 0.04); 1000 mg: −0.7% vs. placebo (*p* < 0.001); HbA1c < 7%: 55.9% vs. 15.2%	LDL-C −11.2 mg/dL; ALT/AST improvement; hs-CRP decline not significant (NS)
NCT06350890 (Phase 3) SYMPHONY-1, DiabetesCare 2026 [15] Level 1b	T2DM inadequately controlled with diet/exercise; 2:1 DB RCT + OLE: HTD1801 1000 mg BID (2000 mg/day) vs. placebo (monotherapy)	N = 407; 28-week OLE; China	HbA1c reduction vs. placebo	1000 mg: LS mean −1.3% vs. placebo −0.6% (LS mean diff −0.7%; 95% CI −0.8 to −0.5; *p* < 0.0001	LDL-C, hs-CRP improvement; sustained HbA1c reduction at 52 weeks
NCT06353347 (Phase 3) SYMPHONY-2, NEJM Evid 2026 [16] Level 1b	T2DM inadequately controlled with metformin; 2:1 DB RCT + OLE: HTD1801 1000 mg BID (2000 mg/day) (add-on to metformin) vs. placebo	N = 549; 24 weeks+ OLE; China	HbA1c reduction vs. placebo (add-on to metformin)	1000 mg: LS mean −1.21% vs. placebo −0.68% (*p* < 0.0001); HbA1c < 7%: 33% vs. 11%	LDL-C/non-HDL-C reduction; GGT improvement; eGFR increase in mild CKD
NCT06415773 (Phase 3) HARMONY, ADA 2026 [17] † Level 1b	T2DM inadequately controlled with metformin; 1:1 DB RCT, head-to-head, both arms on background metformin: HTD1801 (add-on) vs. dapagliflozin (add-on)	N = 369; 24 weeks; China	HbA1c non-inferiority vs. dapagliflozin (margin 0.4%)	1000 mg: HbA1c −1.12% vs. dapagliflozin −0.93% at week 24 (within-group changes); met pre-specified non-inferiority margin of 0.4%; exploratory between-group LS mean difference −0.20% (95% CI −0.37 to −0.03; sponsor-reported *p* < 0.001, not yet independently verified)	LDL-C improvement favoring HTD1801 (magnitude not yet peer-reviewed); eGFR increased with HTD1801 (within-group), with no comparable increase for dapagliflozin (formal between-group comparison not reported); lower statin initiation; serious adverse events: 3.8% (HTD1801) vs. 4.4% (dapagliflozin) [17]
ChiCTR2500110932 (Phase 1b) Mai et al. [18] Level 2b	T2DM + MASLD; dose-escalation (treatment-naïve) + metformin add-on cohort; DB RCT	N = 48; 4 weeks; China	Safety/tolerability; PK of berberine and UDCA	Well tolerated; no SAEs; berberine saturation kinetics, UDCA linear kinetics	Dose-dependent improvement in glucose, lipids, liver enzymes; in metformin cohort (1000 mg) FPG −1.271 vs. +0.423 mmol/L, LDL-C −24.1 vs. +11.2 mg/dL
NCT03381287 (Phase 1b/2a) Di Bisceglie et al. [19] Level 2b	Hypercholesterolemia (LDL-C > 2.59 mmol/L), overweight/obesity; dose-escalation (3:1 active:placebo); DB RCT (monotherapy)	N = 50; 4 weeks; Australia	Safety/tolerability; PK of berberine and UDCA	Well tolerated at all doses incl. 2000 mg/day; no significant AEs; dose-dependent LDL-C reduction	At highest dose (2000 mg/day) vs. placebo at day 28: total cholesterol −8.2% (*p* = 0.0004), LDL-C −10.4% (*p* = 0.0006); no significant change in TG or HDL-C

Footnote: Evidence levels assigned according to the Oxford Centre for Evidence-Based Medicine (CEBM) 2011 Levels of Evidence for therapy/prevention questions [20]. Level 1b, individual randomized controlled trial with adequate blinding and control; Level 2b, individual trial with methodological limitations (e.g., small sample size, non-efficacy primary endpoint). Levels reflect study design and do not by themselves indicate publication status; the trial marked † was available only as a conference presentation or sponsor press release at the time of this review and should be considered preliminary pending full peer-reviewed publication. Unless explicitly labeled a between-group (LS mean) difference, the value shown for HTD1801 and for the comparator arm are each within-group changes from baseline; formal between-group treatment differences are reported only where so labeled. ‘MRI response’ (Harrison et al.) denotes the proportion of patients achieving ≥30% relative reduction in MRI-PDFF from baseline. LDL-C values originally reported in mmol/L (Mai et al. [18]) have been converted to mg/dL (×38.67) for consistency with the other absolute-change value in this table; the relative percentage change reported by Di Bisceglie et al. [19] is retained in its original format as reported in the source publication. Abbreviations: ALT, alanine aminotransferase; AST, aspartate aminotransferase; BID, twice daily; CKD, chronic kidney disease; DB, double-blind; eGFR, estimated glomerular filtration rate; FPG, fasting plasma glucose; GGT, gamma-glutamyl transferase; HbA1c, glycated hemoglobin; HDL-C, high-density lipoprotein cholesterol; HOMA-IR, homeostatic model assessment of insulin resistance; hs-CRP, high-sensitivity C-reactive protein; LDL-C, low-density lipoprotein cholesterol; LS, least-squares (mean); MASH, metabolic dysfunction-associated steatohepatitis; MASLD, metabolic dysfunction-associated steatotic liver disease; OLE, open-label extension; PK, pharmacokinetics; RCT, randomized controlled trial; SAEs, serious adverse events; T2DM, type 2 diabetes mellitus; UDCA, ursodeoxycholic acid.

**Table 2:** Evidence-gap map of HTD1801 combination strategies. Head-to-head comparator data (e.g., HARMONY vs. dapagliflozin) are distinguished throughout from true combination-therapy data, as a comparator trial does not itself establish the safety or efficacy of concurrent use.

Drug Class	Mechanistic Rationale	Clinical Evidence for Combination	Overall Evidence Level
Metformin	(a) Partial AMPK overlap	Clinical: SYMPHONY-2 Phase 3 RCT—HbA1c (LS mean −1.21% vs. −0.68% placebo, add-on to metformin, *p* < 0.0001), LDL-C/non-HDL-C reduction, and GGT improvement	Clinical (Phase 3)
GLP-1RA (semaglutide, tirzepatide)	(a) TGR5-GLP-1 non-overlap	Preclinical only (ADA 2026) [51]; no human combination RCT	Preclinical
Statins	(a) LDLR/PCSK9 non-overlap	HTD1801 monotherapy signal only; no combination RCT	Preclinical
SGLT2i (dapagliflozin)	(b) Downstream convergence	HARMONY head-to-head RCT (comparator only, not combination; both arms on background metformin)	Comparative only
PPAR Agonists (lanifibranor)	(b) Partial AMPK- PPAR overlap	HTD1801and PPAR agonists (e.g., lanifibranor) each have independent monotherapy data; no combination or head-to-head data exist between them	Mechanistic only
Resmetirom	(b) THRβ-mediated pathway, distinct from AMPK/TGR5	No combination or head-to-head data exist	No direct evidence

Abbreviations: AMPK, AMP-activated protein kinase; AST, aspartate aminotransferase; BUDCA, berberine ursodeoxycholate; CKM, cardiovascular–kidney–metabolic syndrome; eGFR, estimated glomerular filtration rate; GLP-1, glucagon-like peptide-1; GLP-1RA, glucagon-like peptide-1 receptor agonist; IL-1β, interleukin-1 beta; IL-6, interleukin-6; LDL-C, low-density lipoprotein cholesterol; LDLR, low-density lipoprotein receptor; LOX-1, lectin-like oxidized LDL receptor-1; MASLD, metabolic dysfunction-associated steatotic liver disease; MASH, metabolic dysfunction-associated steatohepatitis; NF-κB, nuclear factor kappa B; NLRP3, NOD-, LRR- and pyrin domain-containing protein 3; PCSK9, proprotein convertase subtilisin/kexin type 9; PPAR, peroxisome proliferator-activated receptor; RCT, randomized controlled trial; SGLT2i, sodium-glucose cotransporter-2 inhibitor; T2DM, type 2 diabetes mellitus; TGR5, Takeda G protein-coupled receptor 5; THRβ, thyroid hormone receptor-β; TNF-α, tumor necrosis factor-alpha; UDCA, ursodeoxycholic acid.

## Data Availability

No new data were created or analyzed in this study. Data sharing is not applicable to this article.

## Abbreviations

ADA	American Diabetes Association
ALT	alanine aminotransferase
AMPK	AMP-activated protein kinase
AST	aspartate aminotransferase
BID	twice daily
BUDCA	berberine ursodeoxycholate
CKD	chronic kidney disease
CKM	Cardiovascular–kidney–metabolic
CYP	cytochrome P450
CYP2D6	cytochrome P450 2D6
CYP3A4	cytochrome P450 3A4
DB	double-blind
eGFR	estimated glomerular filtration rate
ER	endoplasmic reticulum
FPG	fasting plasma glucose
FXR	farnesoid X receptor
GGT	gamma-glutamyl transferase
GLP-1	glucagon-like peptide-1
GLP-1RA	glucagon-like peptide-1 receptor agonist
GLUT4	glucose transporter type 4
HbA1c	glycated hemoglobin
HDL-C	high-density lipoprotein cholesterol
HOMA-IR	homeostatic model assessment of insulin resistance
hs-CRP	high-sensitivity C-reactive protein
IL-1β	interleukin-1 beta
IL-6	interleukin-6
LDL-C	low-density lipoprotein cholesterol
LDLR	low-density lipoprotein receptor
LOX-1	lectin-like oxidized LDL receptor-1; LS, least-squares (mean)
MASH	metabolic dysfunction-associated steatohepatitis
MASLD	metabolic dysfunction-associated steatotic liver disease
MRI-PDFF	magnetic resonance imaging proton density fat fraction
NF-κB	nuclear factor kappa B
NLRP3	NOD-, LRR- and pyrin domain-containing protein 3
OLE	open-label extension
PCSK9	proprotein convertase subtilisin/kexin type 9
PGC-1α	peroxisome proliferator-activated receptor gamma coactivator 1-alpha
PK	pharmacokinetics
PPAR	peroxisome proliferator-activated receptor
RCT	randomized controlled trial
SAEs	serious adverse events
SGLT2i	sodium-glucose cotransporter-2 inhibitor
T2DM	type 2 diabetes mellitus
TGR5	Takeda G protein-coupled receptor 5
THRβ	thyroid hormone receptor beta
TNF-α	tumor necrosis factor-alpha
UDCA	ursodeoxycholic acid
